# Understanding Social Behaviour in a Health-Care Facility from Localization Data: A Case Study

**DOI:** 10.3390/s21062147

**Published:** 2021-03-18

**Authors:** Gloria Bellini, Marco Cipriano, Sara Comai, Nicola De Angeli, Jacopo Pio Gargano, Matteo Gianella, Gianluca Goi, Giovanni Ingrao, Andrea Masciadri, Gabriele Rossi, Fabio Salice

**Affiliations:** 1 Alta Scuola Politecnica (Politecnico di Milano and Politecnico di Torino), 20133 Milano, Italy; gloria.bellini@asp-poli.it (G.B.); marco.cipriano@asp-poli.it (M.C.); nicola.deangeli@asp-poli.it (N.D.A.); jacopopio.gargano@asp-poli.it (J.P.G.); matteo.gianella@asp-poli.it (M.G.); gianluca.goi@asp-poli.it (G.G.); gabriele.rossi@asp-poli.it (G.R.); 2 Dipartimento di Elettronica, Informazione e Bioingegneria, Politecnico di Milano, 20133 Milano, Italy; andrea.masciadri@polimi.it (A.M.); fabio.salice@polimi.it (F.S.); 3 Cooperativa La Meridiana, 20900 Monza, Italy

**Keywords:** ambient assisted living, data-driven design, social behaviour prediction, social wellness assessment

## Abstract

The most frequent form of dementia is Alzheimer’s Disease (AD), a severe progressive neurological pathology in which the main cognitive functions of an individual are compromised. Recent studies have found that loneliness and living in isolation are likely to cause an acceleration in the cognitive decline associated with AD. Therefore, understanding social behaviours of AD patients is crucial to promote sociability, thus delaying cognitive decline, preserving independence, and providing a good quality of life. In this work, we analyze the localization data of AD patients living in assisted care homes to gather insights about the social dynamics among them. We use localization data collected by a system based on iBeacon technology comprising two components: a network of antennas scattered throughout the facility and a Bluetooth bracelet worn by the patients. We redefine the Relational Index to capture wandering and casual encounters, these being common phenomena among AD patients, and use the notions of Relational and Popularity Indexes to model, visualize and understand the social behaviour of AD patients. We leverage the data analyses to build predictive tools and applications to enhance social activities scheduling and sociability monitoring and promotion, with the ultimate aim of providing patients with a better quality of life. Predictions and visualizations act as a support for caregivers in activity planning to maximize treatment effects and, hence, slow down the progression of Alzheimer’s disease. We present the Community Behaviour Prediction Table (CBPT), a tool to visualize the estimated values of sociability among patients and popularity of places within a facility. Finally, we show the potential of the system by analyzing the Coronavirus Disease 2019 (COVID-19) lockdown time-frame between February and June 2020 in a specific facility. Through the use of the indexes, we evaluate the effects of the pandemic on the behaviour of the residents, observing no particular impact on sociability even though social distancing was put in place.

## 1. Introduction

In the last decades, the rapid advancements and new breakthroughs in the medical field combined with the rising awareness towards healthy life practices have allowed people in developed and developing countries to live longer than ever before. On the other hand, declining fertility rates have been observed in many areas of the world, with highly developed countries reporting the lowest numbers. As a consequence, many societies are currently experiencing a steady increase in the age of their population. In 1990, only 6% of the world population was aged 65 years or over, 10% in 2019 and by 2050 the number is projected to rise to 15%, potentially surpassing that of adolescents, according to the United Nations forecast [1].

The ageing population phenomenon comes with a multitude of brand new problems that still need to be properly addressed, impacting areas from labour markets and economic growth to housing and migration [2]. In the healthcare sector, requests for assistance, which are already on the rise and having an impact on hospitals and other medical infrastructures worldwide, will further intensify, possibly leading to a shortage in service availability.

### 1.1. Alzheimer’s Disease

One of the leading causes of dependency and disability in the elderly is dementia, a chronic, degenerative disease that affects memory, visuospatial abilities, domain and functional cognition, attention, and problem-solving capabilities. Although dementia used to be a relatively rare disease in the past, it has emerged over the last 50 years as one of the most critical issues faced by the developed world. Nowadays the number of affected people is rather large: 5–8% of people aged 60 and over suffer from dementia, rising to 50% when considering people over 85 [3].

Alzheimer’s disease (AD) is the most frequent form of dementia, accounting for about 50% to 80% of all dementia cases [4]. AD can be defined as a severe progressive neurological pathology in which the main cognitive functions of an individual are compromised. It usually starts slowly and gradually worsens over time, with the typical life expectancy following diagnosis being three to nine years [5]. As the condition progresses, the deterioration hinders independence, with subjects eventually unable to perform basic everyday activities without proper help and supervision [6]. At the same time, AD patients are often subject to memory loss of significant life events, which causes a progressive withdrawal from family and society. The many negative effects of the disease significantly affect the quality of life of the subjects and the people close to them, posing a burden both from an economical and from an emotional perspective. The causes of AD are still uncertain, although factors such as ageing and genetics appear to be the main risk factors; moreover, the disease targets women more often than men.

So far, medical sciences have not been able to find an effective treatment to halt or reverse AD progression. Furthermore, recent studies have found that loneliness and living in isolation are likely to cause an acceleration in the cognitive decline associated with AD [7]. Engaging AD patients in social activities and strong connections could therefore be crucial to delay their cognitive decline, preserving their independence, and providing a good quality of life for as long as possible.

### 1.2. Case Study: *Il Paese Ritrovato*

Ambient assisted living (AAL) technologies can improve the assistance of AD patients in innovative healthcare facilities. These structures often share many common features—design inspired to small towns with common open spaces and functional facilities, medium-high independence of patients, *camouflaged* caregivers to have low impact on the everyday life of the residents, and the predisposition of sensory areas dedicated to patients’ reminiscence [8,9]. In the US and Europe, there are a few innovative healthcare facilities and a representative example of them is *Il Paese Ritrovato*.

*Il Paese Ritrovato* [10] is an AD assisted care home in the form of a village, opened in 2018 and managed by La Meridiana [11]. It is the first village dedicated to the treatment of AD ever built in Italy and one of the most innovative Ambient Assisted Living (AAL) projects in Europe. It was built taking the Dutch Hogeweyk village [12] as a reference.

**Figure 1:**
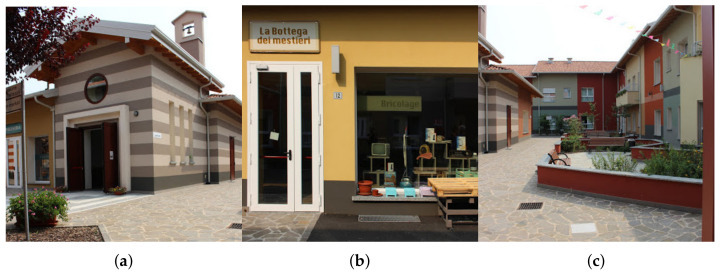
*Il Paese Ritrovato*. (**a**) Village chapel. (**b**) Bricolage lab. (**c**) Piazza.

The complex hosts 64 patients, divided into apartments with 8 private rooms each. All the apartments are equipped with common areas such as a kitchen, a living room, and a TV area, where social interaction takes place. Outside of the apartments, the atmosphere of an actual town is recreated. The central building, which is the pulsing heart of the social life in the village, comprises a theatre, a gym, a church, a hairstylist, a cafeteria, a minimarket, a haberdashery, and a bricolage lab (see Figure 1), where all the professionals running the businesses are in reality nurses specialized in dealing with AD patients—this role-play helps to recreate the safe and stimulating environment typical of a small village. The facility also provides a multitude of large, open, green areas, such as a park and a vegetable garden. Moreover, several brain stimulation activities and therapies are offered in specialized rooms enhancing memory recollection through the sense of smell and sight.

The village also features a plethora of interactive technological devices and appliances. The lighting system can be softened or intensified according to the patient’s will or necessity. Televisions are equipped with smart webcams able to identify facial expressions and identify the emotions of patients, thus reacting to it and possibly adapting the displayed content accordingly. Beds are equipped with pressure sensors located beneath the mattress, which can detect whether a patient is lying or sitting on it. Most importantly, a localization system, introduced by Masciadri et al. [13], allows to track the position of the patients through the use of multiple Bluetooth antennas scattered around the facility and Bluetooth wristbands are worn by the patients. One of the critical issues of the system is the tendency of the patients to reject the wristbands. This problem has been addressed by CLONE [14], an Alta Scuola Politecnica project that focused on the design of comfortable Bluetooth wristbands to avoid stress and rejection.

The typical resident of *Il Paese Ritrovato* is a person diagnosed with mild to moderate AD, in the worst cases resulting in spontaneous confabulation, temporal and spatial context confusion, and personality and behavioural changes leading to occasional delirium [15]. All residents are characterized by a fair level of self-reliance and independence, and can move around the village autonomously. Despite their condition, residents usually establish strong bonds with caregivers, who play a fundamental role in their daily routines, including eating together and conducting social activities.

### 1.3. Supporting AD Patients: State of the Art

Alzheimer’s disease’s complexity is significant, and it is unlikely that any drug or other intervention will successfully treat it. Current approaches focus on helping people maintain mental functions, manage behavioural symptoms and slow down the symptoms of the disease [16]. Typically, AD patients are treated in dedicated facilities in one of the following ways [17]:

- Retirement housing, appropriate for individuals in early-stage Alzheimer’s.
- AAL, which offers a combination of housing, meals, supportive services and health care.
- Nursing homes, which provide around-the-clock care and long-term medical treatment.
- Alzheimer’s special care units, most often cluster settings in which persons living with dementia are grouped on a floor or a unit within a larger residential care building.
- Continuing care retirement communities, which provide different levels of care (independent, AAL and nursing home) based on individual needs.

In all of the aforementioned approaches, AD is treated through medical drugs and the patient lives as if it were hospitalized. The treatment deprives the patient of social interactions, evoking a sense of loneliness and undermining their sociability.

The use of sensors and new technologies to support patients and operators inside these assisted care facilities have been already studied in the literature. The first research of this kind has been published by Doshi et al. [18] who studied the effectiveness of the implementation of a localization system in supporting the activities of caregivers in a nursing facility.

RSSI (Received Signal Strength Indicator) based methodologies used to estimate a human position in an indoor localization system can be divided into three categories: proximity detection, scene analysis, and trilateration. The proximity detection methods have been selected for *Il Paese Ritrovato* since this facility required a low cost, high reliable, coarse-grained localization system and these methodologies are indeed the simplest in terms of implementation.

In their paper [13], Masciadri et al. leverage localization data to assess the physical well-being of an individual. In particular, they measure the physical activity by defining a Movement index, which computes an estimation of the quality and quantity of the movements that a patient performs during a day with respect to the expected movement for the specific patient and taking into account the weather conditions.

Even if the available literature on the usage of localization sensors to support AD care has been mostly directed at mobility assessment [19], the well-being of a person is affected also by his/her social activities. According to Levasseur et al. [20], social activity can be defined as being with others, interacting with others and participating in common activities. However, only being with others and participating in social activities can be inferred using localization data. For this purpose, Masciadri et al. introduce several indexes to estimate the social activity of a person [13]: the Isolation Index (IsI) is a measure of the social dimension of individual wellness that considers the closeness of other persons. In a healthcare facility, it is also important to take into account the average tendency of a person to be in the company of caretakers: at this aim the concept of Independence index (IdI) has been introduced. Finally, the relational index (RI) estimates the strength of the relationship between two persons quantifying the amount of time that they spend together in a given day.

*Il Paese Ritrovato* uses a different, innovative approach, promoting physical activity and socialization other than only medicines to improve the quality of life of the residents and to slow down the progression of the disease. According to Myuri Ruthirakuhan et al. [21], health benefits attributed to physical activity are numerous and well known.

To encourage the patients to perform physical activities and, more in general, to have a healthy and social lifestyle, it is possible to leverage music and many other activities, such as pet and art therapy [22,23]. These activities play a crucial role in enabling people with AD to live a life as satisfying as possible, allowing them to pursue their hobbies and interests, creating immediate pleasure, restoring dignity and enabling friendships [24]. It has been shown that high social engagement reduces the rate of cognitive decline by 91% [25]. On the contrary, both actual social isolation, including having a small social network and participating in few activities with others, and perceived social isolation, that is, feeling lonely, are robustly associated with AD progression and cognitive decline [26].

### 1.4. Purpose and Contributions

This work leverages the possibility of monitoring the localization of AD patients inside assisted care homes through a network of distributed sensors and antennas to extract patterns, gather insights, and model social behaviours through statistical and machine learning techniques.

Typically, sociability is assessed through questionnaires and related indexes, combining sociology and medical sciences. However, as we were starting from objective localization data, we leverage a completely different approach: we start from data and define objective indices to assess and quantify either the sociability among dwellers or the degree of popularity of a place. To the best of our knowledge, there are no examples of similar methodologies available in literature.

Bluetooth Low Energy (BLE) tags, initially in the form of bracelets, were used for tracking the guests throughout the facility. The wearable device was modified several times during the trial, due to battery consumption and/or IP level (dwellers would wear bracelets also while showering). An always growing percentage of users—initially around 50%—tended to abandon these devices, throwing them away, burying them in the gardens, and flushing them in the toilet. The solution for this set of hosts included disguising them through brooches, sewing them into clothing, inserting them into shoe soles—note that this latter solution requires more investigation due to the mechanical fragility of the inserted tags and data loss due to beacon signal masking. A solution allowing for large improvement, even in terms of accuracy of the localization system, was given by multi-tagging—multiple tags applied to the same individual in different and hidden places. This approach, though being more expensive than the single-tag one, also in terms of maintenance, has also implicitly introduced a mechanism of “human faults” tolerance and allowed to monitor all guests for a relatively long period, also thanks to the particular attention of the caregivers.

Our purpose is to analyze these localization data, build visualization tools to observe and comprehend the indexes of sociability and popularity introduced in previous works [13,27] and design predictive models for their estimation over time.

As such, we present visual tools and methodologies based on these indexes for the identification of clusters of patients, isolation, and close friendships in a community.

We propose the Community Behaviour Prediction Table, a visual tool leveraging predictive models to support caregivers in organizing activities, along with extensive analyses and examples on how to leverage this tool to schedule state-of-the-art, AD-designed activities. This tool can prove to be valuable for caregivers as the compatibility of an activity with the location it is held at and the social profiles of the participants engaged are crucial to guarantee its therapeutic benefits.

Finally, we present an analysis of our specific case study during the lockdown period due to COVID-19 to verify the actuation of preventive social distancing measures, showing one of the many possible applications of the sensors, indexes, and tools.

## 2. Materials and Methods

In this Section we present both descriptive and predictive tools allowing for analysis. These tools are built starting from the localization data of patients. Therefore, a fundamental requirement of the system we interface with is the capability of collecting localization data ensuring patients’ privacy. The system of the considered case study was built by Masciadri et al. [13] and consists of a coarse-grained localization module based on iBeacon technology [28]. We will consider their implementation as a reference.

### 2.1. Sensors and Data

**Figure 2:**
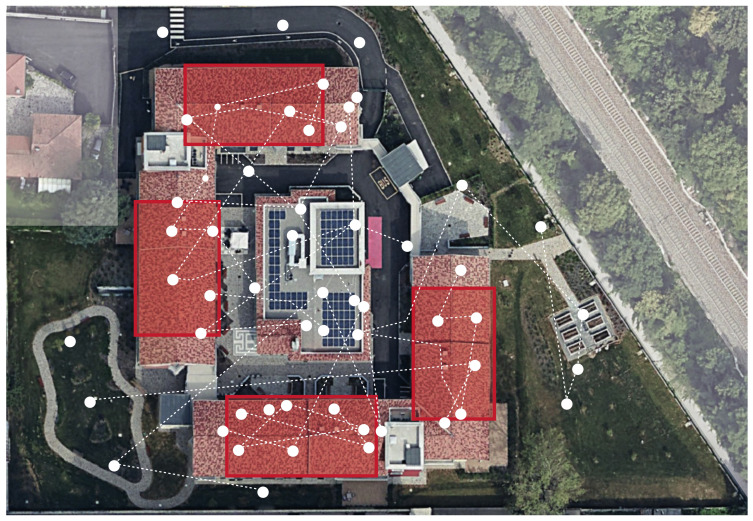
The network of antennas at *Il Paese Ritrovato*. The red areas are the private places of the structure while the white ones denote the areas outside the facility. The rest are the public areas of the facility. Notice that the top north street is the car access, so it is forbidden to dwellers.

For the system to work, patients must wear the bracelet at all times and agree to be localized throughout the facility they are hosted in. iBeacon technology has multiple benefits—a long battery life of about 6 months, low complexity and low cost. A network of antennas scattered across the village allows localizing the patients by employing the Received Signal Strength Indicator (RSSI) methodology, assigning each patient to the closest antenna. To avoid frequent position shifts between near antennas and to increase the granularity of the data, providing a higher privacy level to the residents, it was decided to divide the antennas into non-overlapping clusters (see Figure 2). Using this approach, the position of a resident in a given timestamp is represented by the closest cluster, instead of the closest antenna. The data is collected every 10 s and it is stored in a database for further inspection and analysis. Thus, this system provides real-time knowledge on the position of residents throughout the facility.

To ensure the privacy of the residents is respected, the data we use for our analysis is anonymized by assigning a random identifier to each resident and by removing all information allowing identification. Residents agreed both to the collection of their data and on their analysis.

In summary, the data extracted by the presented system consists of the position of each resident (in terms of clusters to which they belong) computed every 10 s. As we collect data every 10 s, the time model we refer to is not continuous but discrete. We define T⊂N as the set of natural numbers from 1 to 8640: T={1,⋯,8640}. *T* represents the points in time during the day when localization data is collected. On top of that, we compute many different metrics, such as the approximate distance walked by each resident per hour, to extract meaningful insights from these data.

### 2.2. Interfacing with the Database

The localization data collected at Alzheimer’s Care home facilities are hosted in a secure, private cloud database on Microsoft Azure. As we are handling private data, we revise Microsoft Azure privacy policy, verify its compatibility with the policies of the facility and protect our database with a strong password and whitelisted IP addresses.

Despite being a very popular query language among computer scientists, we decide to implement a database library acting as a wrapper of the MySQL database to make it easily interactive through high level Python objects and methods. This allows increasing productivity when writing data analysis scripts. Using Python to develop the wrapper also provides a powerful and expressive language. As an example, many functionalities provided by the library are implemented to account for many different time granularities such as day, hour, and minute. This allows us to later exclusively focus on the statistical and machine learning part of the problem and easily test new configurations without having to deal with cumbersome implementation details.

Finally, we try to provide a framework that is as general as possible, that is, compatible with a generic nursing home that has access to a database containing localization data about their patients. As an example, our library can be used in locations having different space configurations and number of individuals than the ones in our case study. As such, the presented library can prove to be useful to data scientists and researchers dealing with similar problems both at Alzheimer’s and other assisted care homes in the world.

### 2.3. Indexes Measuring the Strength of Relationships

Building social well-being is crucial for Alzheimer’s Care Home Facilities. Being socially active by spending time with others allows people to feel less angry, lonely, and disconnected. Gaining insights on the social dimension can therefore contribute to the overall assessment of patients’ well-being.

Social activities can be distinguished in: being with others, interacting with others, and participating in common activities. While interaction detection requires dedicated devices and sophisticated techniques related to the field of Artificial Intelligence leveraging Computer Vision and Natural Language Processing techniques, the mere involvement in social activities can be inferred through the use of localization data. With the data at our disposal, we have information regarding which user is in a certain place at a certain time. We hereby present a measure of sociability introduced in [13], and extend its definition to study the behaviour of single individuals and of the entire community.

#### 2.3.1. The Relational Index

The Relational Index (RI), introduced by Masciadri et al. [13] aims to estimate the relation between two individuals measuring the amount of time they spend together in a day. The RI is defined for a couple of individuals *i* and *j* and it is calculated by counting the number of times the two individuals are located under the same place throughout a certain day. The RI for individuals *i* and *j* on day *d* is:

$$\begin{array}{cc} {\tilde{RI}}_{i,j}^{d}=\frac{\sum_{t=1}^{T}V_{i,j}^{d}\left(t\right)}{\left|T\right|},\;\mathrm{where} & V_{i,j}^{d}\left(t\right)=\left\{\begin{array}{cc} 1 & \mathrm{if}\;i\;\mathrm{and}\;j\;\mathrm{are}\;\mathrm{in}\;\mathrm{the}\;\mathrm{same}\;\mathrm{place}\;\mathrm{on}\;\mathrm{day}\;d\;\mathrm{at}\;\mathrm{time}\;t\\ 0 & \mathrm{otherwise}, \end{array}\right.\\ \; & t\in T=\{1,\cdots ,8640\},\;\left|T\right|\;\mathrm{being}\;\mathrm{the}\;\mathrm{cardinality}\;\mathrm{of}\;T. \end{array}$$

The Relational Index assumes values in the interval [0,1], 0 being the two very socially distant and 1 being completely socially close. For instance, ${\tilde{RI}}_{2,3}^{20/12/2020}=0.25$ indicates that on 12 December 2020, individuals 2 and 3 spent 25% of the time in the same room.

It must be noted that a pair of individuals cannot have a relational index equal to 1 since they are required to be in their (single) room for the night and we observed that also during the day they spend a considerable amount of time in their room by themselves.

The ${\tilde{RI}}_{i,j}^{d}$ index does not take into account the meaningfulness of the interaction: individuals that suffer from wandering [29] may have a high relational index just because they often walk near other dwellers. To solve this problem, we introduce the concept of continuity of interaction by modifying $V_{i,j}^{d}\left(t\right)$. We define $M_{i,j}^{d,k}\left(t\right)$ as the median of $V_{i,j}^{d}\left(t\right)$ on a centred moving median of size *k*. We set *k* to 5 to remove all the interactions that last less than 20 s, thus considering them as fortuitous contacts that do not contribute to the socialization between dwellers. For simplicity reasons, we copy the first $\frac{k}{2}$ values since it is not possible to center a moving median of size *k* on them.

However, this definition cannot be used to filter out short interactions: the larger the rolling median window size, the greater the time interval in which a chance encounter or accidental circumvention can be confused with a significant interaction or non-interaction.

For instance, with respect to Table 1, let us assume $V_{i,j}^{d}\left(t\right)$ to be the vector in the first line, $M_{i,j}^{d,11}\left(t\right)$ to be the moving median of size 11 in the second line, and $M_{i,j}^{d,5}\left(t\right)$ to be the moving median of size 5 in the third line.

The centred moving median with size 11 $M_{i,j}^{d,11}\left(t\right)$ could aggregate the interactions present at the beginning of the vector with the meaningful interaction at the end. Instead, by using a moving median with a smaller size $M_{i,j}^{d,5}\left(t\right)$ the resulting vector is more representative of the actual interaction.

To solve this problem, we use a separate filter to discard the interactions in $M_{i,j}^{d,k}\left(t\right)$ shorter than 60 s, and consider only the longer ones to count towards the index of sociability.

As a result, we obtain ${\hat{V}}_{i,j}^{d}\left(t\right)$, redefining the relational index as:

$$\begin{array}{c} RI_{i,j}^{d}=\frac{\sum_{t=1}^{T}{\hat{V}}_{i,j}^{d}\left(t\right)}{\left|T\right|}. \end{array}$$

As in [27], we extend the definition of the RI to capture the sociability of an individual dweller. Considering a user as active on day *d* when we can track their position throughout day *d*, we define the Individual RI as:

$$\begin{array}{cc} RI_{i}^{d}=\frac{\sum_{j\in A^{d}\setminus \left\{i\right\}}{RI_{i,j}^{d}}^{2}}{|A^{d}\setminus \left\{i\right\}|},\;\mathrm{where}\; & A^{d}\;\mathrm{is}\;\mathrm{the}\;\mathrm{set}\;\mathrm{of}\;\mathrm{active}\;\mathrm{users}\;\mathrm{on}\;\mathrm{day}\;d,\\ \; & A^{d}\setminus \left\{i\right\}\;\mathrm{is}\;\mathrm{the}\;\mathrm{set}\;\mathrm{of}\;\mathrm{active}\;\mathrm{users}\;\mathrm{on}\;\mathrm{day}\;d\;\mathrm{without}\;\mathrm{user}\;i. \end{array}$$

Since the RI for an individual over the entire day is computed as the mean of squares, spending a certain amount of time with a single person is valued more than spending the same amount of time cumulatively with more people. For instance, an individual spending 6 h in their kitchen together with another person should be considered more sociable than one spending 30 min at the bar with 12 people. Yet, if the Individual RI were to be a linear mean, then the second individual would be considered as sociable as the first one since for both of them $\sum_{j\in A^{d}\setminus \left\{i\right\}}RI_{i,j}^{d}$ would be equal to 0.25. On the other hand, using the mean of squares leads the relational index of the first individual to be twelve times higher than the one of the second. As a consequence, we decided to use the mean of squares, as we believe meaningful and strong bonds are more valuable than occasional presence in crowded areas.

To capture the sociability of the whole community, we extend the RI to a group of individuals. We define the Community RI as:

$$\begin{array}{cc} RI^{d}=\frac{\sum_{i\in A^{d}}RI_{i}^{d}}{|A^{d}|},\;\mathrm{where}\;A^{d} & \;\mathrm{is}\;\mathrm{the}\;\mathrm{set}\;\mathrm{of}\;\mathrm{active}\;\mathrm{users}\;\mathrm{on}\;\mathrm{day}\;d. \end{array}$$

The $RI^{d}$ indicator combining the sociability of all dwellers could reveal some useful insights. For instance, caregivers could look at the long-term trend of this indicator to understand if there is an overall process of isolation going on. This indicator is therefore similar to the Individual Relational Index, but considers the whole structure as a whole.

#### 2.3.2. Time-Specific Relational Index

Analyzing sociability is a hard task, especially when it comes to AD patients. We extend the definition of the RI to time-specific measures to group together the different times of the day, that is, morning, noon and afternoon.

We define the Time-Specific RI for two individuals as their RI limited to a specific interval of time during the day:

$$\begin{array}{cc} RI_{i,j,\hat{T}}^{d}=\frac{\sum_{t\in \hat{T}}V_{i,j}^{d}\left(t\right)}{|\hat{T}|},\;\mathrm{where}\;V_{i,j}^{d}\left(t\right) & =\left\{\begin{array}{cc} 1 & \mathrm{if}\;i\;\mathrm{and}\;j\;\mathrm{are}\;\mathrm{in}\;\mathrm{the}\;\mathrm{same}\;\mathrm{place}\;\mathrm{on}\;\mathrm{day}\;d\;\mathrm{at}\;\mathrm{time}\;t\\ 0 & \mathrm{otherwise}, \end{array}\right.\\ \hat{T}\subseteq T & \;\mathrm{is}\;\mathrm{the}\;\mathrm{considered}\;\mathrm{time}\;\mathrm{of}\;\mathrm{the}\;\mathrm{day}. \end{array}$$

Considering the relationship $RI_{i,j,\hat{T}}^{d}$ between individuals *i* and *j* on day *d* during interval $\hat{T}$, we define the contribution this relationship has on the overall sociability of *i* $RI_{i}^{d}$ as $\Delta_{i,j,\hat{T}}^{d}=\left(RI_{i,j,\hat{T}}^{d}-RI_{i}^{d}\right)$. Thus, we define the Time-Specific Adjusted RI for individual *i* in relation to individual *j* as:

$${\hat{RI}}_{i,j,\hat{T}}^{d}=RI_{i,j,\hat{T}}^{d}+\gamma_{i,j,\hat{T}}^{d}\Delta_{i,j,\hat{T}}^{d},$$

where $\Delta_{i,j,\hat{T}}^{d}$ serves as a way to reward (penalize) the relationship between *i* and *j* if it contributed positively (negatively) to the Individual RI of *i*, and $\gamma_{i,j,\hat{T}}^{d}$ is a multiplication term that ensures that the new index ${\hat{RI}}_{i,j,\hat{T}}^{d}$ assumes values in the interval [0,1] and is defined as:

$$\begin{array}{c} \gamma_{i,j,\hat{T}}^{d}=\left\{\begin{array}{cc} \left(1-RI_{i,j,\hat{T}}^{d}\right) & \mathrm{if}\;\Delta_{i,j,\hat{T}}^{d}\geq 0\\ RI_{i,j,\hat{T}}^{d} & \mathrm{otherwise}. \end{array}\right. \end{array}$$

The newly defined index ${\hat{RI}}_{i,j,\hat{T}}^{d}$ measures the social interactions between individuals *i* and *j* taking into consideration the Individual RI of individual *i* in the term $\Delta_{i,j,\hat{T}}^{d}$.

For instance, if $RI_{i,j,morning}^{d}=0.3$ and $RI_{i}^{d}=0.2$, it means that the relationship individual *i* had with dweller *j* in the morning had a positive impact on the overall sociability of dweller *i* over day *d*, resulting in ${\hat{RI}}_{i,j,morning}^{d}=0.37$. On the contrary, if $RI_{i,j,morning}^{d}=0.05$ it means that the relationship between the two did not have a positive impact on the overall sociability of *i* on day *d*, and we therefore penalize it by computing ${\hat{RI}}_{i,j,morning}^{d}=0.0425$.

Furthermore, we introduce the Individual Time-Specific Adjusted RI for individual *i*, as the average of his/her Time-Specific Adjusted RI with the rest of the community, during a specific time of the day:

$$\begin{array}{cc} {\hat{RI}}_{i,\hat{T}}^{d}=\frac{\sum_{j\in A^{d}\setminus \left\{i\right\}}{\hat{RI}}_{i,j,\hat{T}}^{d}}{|A^{d}|-1},\;\mathrm{where}\;A^{d} & \;\mathrm{is}\;\mathrm{the}\;\mathrm{set}\;\mathrm{of}\;\mathrm{active}\;\mathrm{users}\;\mathrm{on}\;\mathrm{day}\;d. \end{array}$$

Finally, we define the Community Time-Specific Adjusted RI as the average Individual Time-Specific Adjusted RI:

$$\begin{array}{c} {\hat{RI}}_{\hat{T}}^{d}=\frac{\sum_{i\in A^{d}}{\hat{RI}}_{i,\hat{T}}^{d}}{|A^{d}|}. \end{array}$$

These indicators, combined with the ones defined in Section 2.3.1, might give insights to caregivers when studying how the sociability of dwellers changes at different times of the day. For instance, a Community Time-Specific Adjusted RI higher in the afternoon than in the morning, is a read flag of an activity scheduling that might be improved, leading to a possible change of the activities organized in the morning to ones that enhance sociability more.

### 2.4. Indexes Assessing the Attendance of Places

Social interactions are often bound to the places where they occur. In our previous work [27] we introduced a measure of the popularity of places, called the Popularity Index. Considering the context of Alzheimer’s Care Home Facilities, our definition of “popular place” can be given in light of this statement—the more a place is frequented, the more it can be considered popular. Without any information on the personality of the residents, we are not able to consider the kind of people present at a certain place, but we have to limit our analysis to quantitative aspects and consider the number of residents.

Based on the popularity of each place, caregivers and staff can easily understand which places are more likely to attract a large crowd and which ones are less visited. Depending on the circumstances, higher or lower levels of popularity may be desired for a given place, which can be achieved by implementing architectural changes and redesigning the space around it. Furthermore, staff may also want to leverage the prediction of the popularity of a place so that they can dynamically adapt the activities schedule to provide residents with a better experience.

#### The Popularity Index

As we wish to measure the popularity of the places of the facility, we leverage the Popularity Index (PI) that is a measure of the attendance of a place since it considers the popularity of a certain place to be positively correlated with the number of people visiting that place. From the previous section, we know the dwellers’ position every 10 s. While this timeframe is suited to evaluate interactions between people, as their duration is limited in time and it is in the order of a few minutes, to evaluate the attendance of a place things are a little different. Being caught for a few minutes inside an area does not necessarily mean that you are attending that place. Assume you have to cross a piazza to reach your destination and this crossing takes you some minutes. In these cases, the piazza cannot be considered an attended place in a strict sense. Thus, to exclude too small timeframes, we resort to adopt a larger granularity to compute this index, taking into account the hourly accesses to a given place.

Let $n_{p}^{d}\left(h\right)$ be the number of detected individuals in place *p* over an hour starting from hour *h* on day *d*. Then, the PI of place *p* on day *d* is defined as:

$$PI_{p}^{d}=\frac{1}{|A^{d}\left||D\right|}\sum_{h\in D}n_{p}^{d}\left(h\right),$$

where *D* is the set of hours in which users are generally awake and $A^{d}$ is the set of active users on day *d*.

This index is the 18-hour averaged accesses to a certain place, normalized by the number of active users: smoothing is obtained through the averaging procedure, the focus is on the parts of the day in which social interactions are relevant, and the normalization makes the index fully objective, avoiding potential biases due to a higher number of tracked individuals.

**Figure 3:**
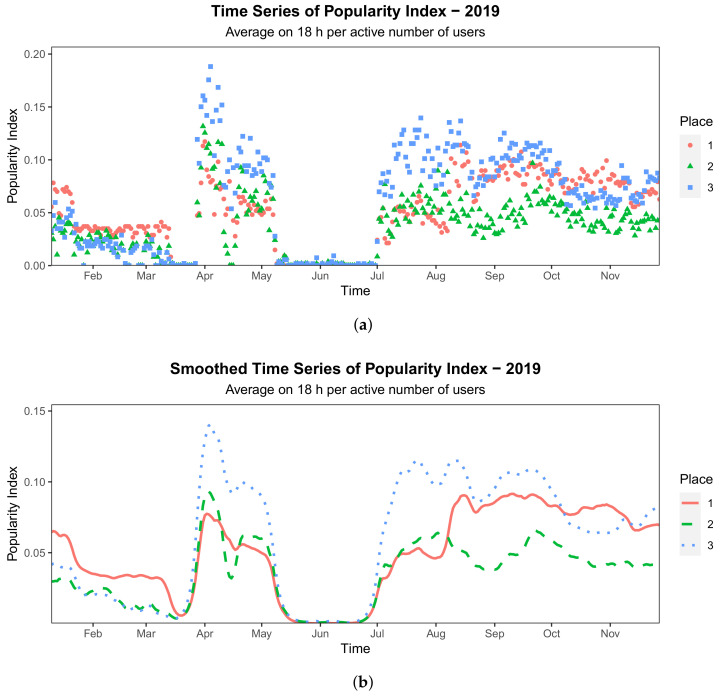
Comparison between computed and smoothed times series of the Popularity Index (PI). (**a**) Before Smoothing. (**b**) After Smoothing.

Once the Popularity Index is defined, we need to find the proper approach to analyze these data. Final aim is to observe different trends over time in the Popularity Index, to distinguish frequented areas and unpopular ones. The approach we rely on is Functional Data Analysis (FDA) [30], a branch of statistics that interprets data as a discretized sample of a continuous function, in a suitable functional space. In this way, we can associate a unique Popularity Index trend to each place and the data will be easily grouped in light of remarkable differences in such trends, even in the case this difference is limited to a single portion of the whole observation period. To resort to Functional Data Analysis, we require continuous data, thus the curves need to be “smoothed”, that is, embedded in a suitable functional space. This procedure is fundamental and can also be beneficial to remove some noise that the raw data exhibit (see Figure 3a). In this framework, a statistic unit, that is, a generic curve $Y_{i}$, is seen as a linear combination of functions:

$$Y_{i}\left(x\right)=\sum_{j=1}^{N}\alpha_{ij}\psi_{j}\left(x\right)+\epsilon_{i},$$

where ${\left\{\psi_{j}\left(x\right)\right\}}_{j=1:N}$ are a set of suitable basis functions, $\epsilon_{i}$ is the curve-related random effect (usually modelled as a zero-mean Gaussian random variable) and coefficients $\alpha_{ij}$ need to be determined.

It is crucial to select the proper basis function. Since the time series do not exhibit any kind of evident periodicity, the fact that data are non-negative and that we require a certain degree of tuning to clean some noise residue, we ended up choosing penalized B-spline basis functions of 3rd order [31]. These type of smoothing allows us to:

- gain some regularity of the function since, at third order, functions are of class $C^{3}$;
- preserve the non-negativeness of the functions since we choose to penalize, through a parameter λ, the first derivative of the smoothed curves, thus letting us free to smooth strong peaks of noise;
- avoid fixing the dimension of the basis space since with this type of splines the dimension is automatically selected to generate curves that blindly follow the original data.

We show a comparison between the curves before and after the smoothing procedure in Figure 3. The resulting curves preserves the main characteristics of the original ones and visibly reduces the residual noise. 

### 2.5. Predictive Tools for the Sociability of the Community

Through an in-depth analysis of the correlation between the social behaviour of individuals measured through the Individual RI and other variables, in [27] we observed that patients’ social behaviour depends on the season, the temperature, the dweller’s bedroom floor, the predisposition to walk on a certain day and the patient’s average hourly walked distance. Given some of these variables, we aim to exploit the observed dependence and predict the social behaviour of the whole community. Together with the popularity index, this allows a better organization of the social activities at the Alzheimer’s Care Home Facilities.

To predict the social behaviour of the community, we choose to build a predictive model for the Community Time-Specific Adjusted Relational Index, defined in Section 2.3.2. Among the correlated variables we choose to exclude the bedroom floor and the average hourly walked distance, being patient-specific. To enrich the predictive power of our model capturing more elements of the environment we assess the correlation between social behaviour and the following two variables: the specific time of the day, that is, morning, noon, afternoon, and the attribute of a day being a workday. After confirming they are correlated, we include them in the variables set for prediction. Therefore, the variables we consider for the prediction of the Community Time-Specific Adjusted Relational Index are the following:

- attribute of a day being a workday;
- season;
- temperature;
- predisposition to walk on a specific day and specific time of the day.

Having considered three different times of the day, for each day we have a piece of data, accounting for a total of about 1000 records as we are considering a time frame of about a year and have 3 records per day. Considering that the relationship between the considered variables and the one to be predicted is unknown and most likely quite complex as we are considering social behaviour, we choose to implement a neural network to model it and accomplish the task of prediction [32]. The task can be modelled as a regression problem and the network structure is hereby briefly described. The input and output consist of a neuron for each aforementioned variable, except for the season, which requires 3 neurons, being a categorical variable. The hidden part of the network consists of 4 layers of 128 neurons each, activated by a traditional Rectified Linear Unit (ReLU) function [33]. The chosen loss function is given by the Mean Squared Error (MSE) and the Adam optimization algorithm [34] is used to perform gradient descent. To measure the performance of the network, we randomly divide the records into two disjointed splits: training (70% of the samples) and test set (remaining 30% of the samples).

### 2.6. Predictive Tools for the Popularity of Places

The data on the movements can be used not only to inspect the social behaviour of the dwellers, but also to examine the attractiveness of the various public places in the facility. In particular, the PI presented in Section 2.4 is a robust measure of the popularity of a place and can be helpful to determine the areas that are more likely to engage dwellers in social activities. Given the dataset, it would be desirable to find correlations between the PI of a place and other variables to ultimately predict its value, so that activity planners, that is, caregivers, can use this information to schedule activities that are more likely to be engaging.

We thus need to find a set of input variables in the dataset that are both strongly correlated with the PI and easy to predict. Examples of potentially relevant input variables are the weather, which can be assumed to be correctly predicted by online forecasters, and the period of the year, which is deterministic. Upon a visual examination of the data in our dataset, we find the following variables to be especially meaningful when predicting the Popularity Index of a given place at a certain time:

- place identifier;
- period of the year;
- period of the day;
- weather;
- temperature.

Due to the nature of the problem, we are mainly interested in public places that are external to the residential buildings such as the gym, the garden, and the café. As we are trying to evaluate the popularity of a certain place when activities are held in that place, we remove from the dataset the records with PI lower than 0.01, which is enough to reasonably assume no event is ongoing in that place at that time. An improvement to this heuristic would be to integrate information on the actual activities held at the facility and extract the relevant data records accordingly.

We model the prediction task as a regression problem where the value of the PI of a given place at a certain time across the year is estimated based on the inputs mentioned above. Since the correlation between input and output is potentially far from trivial, we opt to train a neural network to automatically extract interesting patterns from data. To do this, we use PyTorch [35], a popular Python machine learning library. Still, we would like to embed into the model as much domain knowledge as we possibly can to improve its performance and introduce regularization. One way to do this is to introduce semantics in the input.

#### 2.6.1. Place Identifier

Using a single scalar value in this case may lead to unwanted behaviours, such as recognizing two places as similar solely based on their identifiers. We thus encode the *n* public places using the so-called one-hot encoding, transforming the identifier of the *i*th place into a vector having *n* cells set to 0, besides the *i*th one that is, instead, set to 1. For instance, assuming *n* equal to 3, place 0, 1 and 2 will be respectively represented by vectors [1,0,0], [0,1,0] and [0,0,1]. The one-hot vector is then preprocessed by the neural network and mapped onto a 3-dimensional latent space capturing relevant features of each place.

#### 2.6.2. Period of the Year

**Figure 4:**
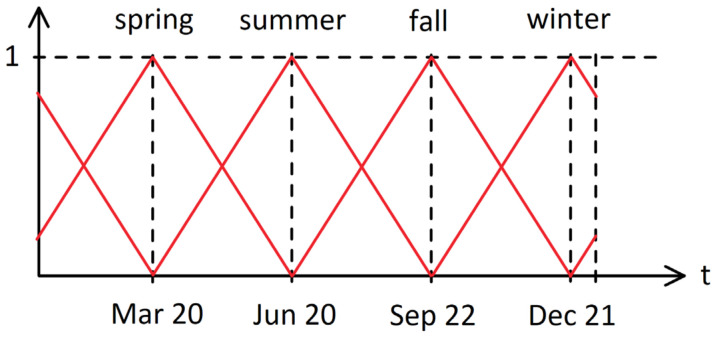
Triangular fuzzy set on the seasons of the year.

We encode the information as a triangular fuzzy set on the seasons of the year, as shown in Figure 4. For example, when considering the winter solstice, the information will be encoded as [0,0,0,1], while the middle of spring, which is equidistant from the spring equinox and the summer solstice, will be encoded as [1/2,1/2,0,0]. The model can thus easily interpolate and generalize on unseen periods of the year. In this way, we also avoid discontinuities that may occur between 31 December and 1 January when using other trivial encodings (e.g., linear mapping).

#### 2.6.3. Hour of the Day

We are interested in the time of the day where dwellers will be awake and active, therefore from 6 A.M. to midnight. We choose to encode this interval of time using a linear mapping onto [0,1], where a generic timestamp *t* (in seconds) corresponds to:

(1) $$t_{enc}=\frac{t-6\times 60\times 60}{24\times 60\times 60-6\times 60\times 60}=\frac{t-21,600}{64,800}.$$

Given this encoding, 6 A.M. corresponds to 0 and 12 P.M. to 1, while all the timestamps belonging to the selected interval of time are encoded to values ∈[0,1]. For instance, 5 P.M. will be encoded as

(2) $${\left\{5\;P.M.\right\}}_{enc}=\frac{17\times 60\times 60-21,600}{64,800}=\frac{61,200-21,600}{64,800}=0.6\bar{1}.$$

In this way, it is possible for the network to predict unseen time instants through simple interpolation. Note that, though it is true that time is circular, it would not be appropriate to consider late night as similar to early morning from a social and physiological perspective.

#### 2.6.4. Weather

We encode the weather using one-hot encoding, taking into account three different types of weather: sunny ([1,0,0]), cloudy ([0,1,0]), and rainy ([0,0,1]).

#### 2.6.5. Temperature

We encode the relevant values, that is, from 0 to 40 °C, onto [0,1] through a linear mapping, as done for the hour of the day.

We generate an input dataset with about 109,000 records. Each piece of data is characterized by the PI, the period of the year, the period of the day, the weather and the temperature of the place the measurement refers to. These samples are then randomly divided into two disjointed splits, training and test set. The training set, which contains 70% of the input records, is then used to train a modular feed-forward neural network [36] with a variable number of layers. We investigate the right number of layers, along with their dimension, through a grid search, testing the performance of each configuration on the test set.

First, we set the properties of the neural network.

- The first layer receives the place id one-hot vector as input, to encode it in a 3-dimensional latent space. The idea behind this choice is to reduce the dimensionality of the place id vector, thus reducing the complexity of the network.
- The second layer takes as input the remaining 9 values from the remaining input variables, along with the 3-dimensional vector encoded by the first layer (representing the place). The output dimension of this second layer varies between 16, 64 and 256 in our grid search.
- The final layer consists of a single output neuron, having a Leaky ReLU activation function at training time and a ReLU activation function at prediction time [33]. This allows avoiding zero-gradient problems when training while correcting negative outputs when inferring, as the PI is always non-negative.

We also apply an early stopping technique to avoid overfitting [37] and train the model in a supervised manner using MSE as loss function and Adam optimizer for gradient descent. The output of the described neural network is the Popularity Index: a floating-point value between 0 and 1, representing the percentage of dwellers expected to be located at a certain place, for a specified hour of the day, and in a certain period of the year, also according to the expected weather and temperature.

The final structure of the network is chosen among 27 different possible configurations through a grid search that considers:

- the hidden layers dimension, with 3 possible values: {16, 64, 256};
- the scaling factor, that is, the factor for which the layer dimension was multiplied by when moving from a layer to the subsequent one, with 2 possible values: {0.5, 1};
- the number of hidden layers, from 1 to 5.

For instance, a hidden layer dimension of 256, a scaling factor of 0.5 and several hidden layers equal to 3 generate the following network structure:

(3) $$9\times 12\times 256\times 128\times 64\to PI_{expected},$$

where each number represents the number of inputs of a layer, while the arrow represents the output of the network.

These grid search parameters aim to obtain a reasonable trade-off between coverage of the space of possible models, which is desirable to find the true optimal configuration, and the computational power we need to train all the possible models. After performing the grid search, the resulting best performing architecture is the following:

(4) $$9\times 12\times 64\times 32\times 16\to PI_{expected},$$

Indeed, we observe that both the simplest and most complex model in our selection perform worse than the best configuration we have found through the grid search, suggesting that our range of considered values is suitable for the problem at hand.

## 3. Results

In Section 2 we have extended and defined new indexes capable of measuring the level of sociability among patients and the level of popularity of spaces within a facility. Here we show how these indexes can be used to provide visual tools for doctors and caregivers to be used in activity scheduling. Our case study is *Il Paese Ritrovato* (see Section 1.2 for further details), where the tracking system is obtained through Bluetooth bracelets. Finally, we analyze data during the first lockdown period due to COVID-19 pandemic (January to June 2020) to show another possible usage of these indexes, not as support in activity planning, but as a monitoring tool for the adoption of social distancing measures.

### 3.1. Predictive Models Evaluation

#### 3.1.1. Community Time-Specific Relational Index

Through the presented network of Section 2.5 we can predict the Community Time-Specific RI for a certain day and specific time of the day-considering if that day is a workday, the season, the temperature, the predisposition to walk on that day-with a Mean Absolute Error of 0.025.

The index is not a definitive and complete measure of the sociability of the community: the sociability is not only given by the time the residents spend together in the same place, but also by the quantity and the quality of interactions they have, which is not measurable with the currently implemented technology. Patients could all be in the same place without socializing with one another and their indexes of sociability could be quite high.

However, this result is still quite significant for our case for two reasons. First, considering the index domain in [0,1] and the scope, a confidence of ±0.025 is enough. Then, most importantly, not all the variables impacting on the sociability of an individual, measured by the RI, are considered since it is practically impossible to know all of them and some are impossible to measure with the current technology, as it is the case for most social and irrational factors.

#### 3.1.2. Popularity Index

Using the best performing network presented in Section 2.6, we can predict the PI of a given place for a certain day and specific hour of the day—considering also the weather, the temperature and the period of the year—with a Mean Absolute Error of 0.012.

The previous considerations for the RI still hold for the PI. Also, in this case, the model’s predictive performance is limited by the quality of the data available since the dwellers tend to lose their bracelets and, even if the dataset has been substantially cleaned beforehand, it is not possible to detect and remove all the outliers.

### 3.2. Community Behaviour Prediction Table

Caregivers at *Il Paese Ritrovato* organize several social activities for the residents. Thanks to these activities residents may pursue their hobbies and interests and engage in social relationships with others. Besides satisfying the residents, these activities are also beneficial since they slow down the progression of the disease: according to Mace [24], social engagement reduces the rate of cognitive decline by 91%.

Today, activity scheduling is only based on the knowledge of caregivers, doctors and psychologists at *Il Paese Ritrovato*. Their technical skills, combined with their deep understanding of the community of residents is crucial to guide activity scheduling. The staff works daily in the assisted care home and knows quite well the activities’ potential and drawbacks, besides having a personal direct relationship with the dwellers. However, this decision process can be enriched further.

Each activity is characterized by specific constraints and desired conditions. For instance, the weather being a hard constraint for gardening; handcrafting being suitable for a small number of participants. The sociability of participants is also a crucial aspect of an activity. An activity requiring people to interact and share ideas may not be suitable for a group comprising very shy individuals not keen on connecting, whereas an activity in which a few participants are involved may be more beneficial when the sociability of the community is low. Moreover, the place in which an activity is held plays an important role. Once hard constraints are met, it could be game-changing to identify a location to attract those individuals who are just swinging by. Furthermore, holding a certain activity in an overlooked place may stimulate the interest of the participants in that place.

**Figure 5:**
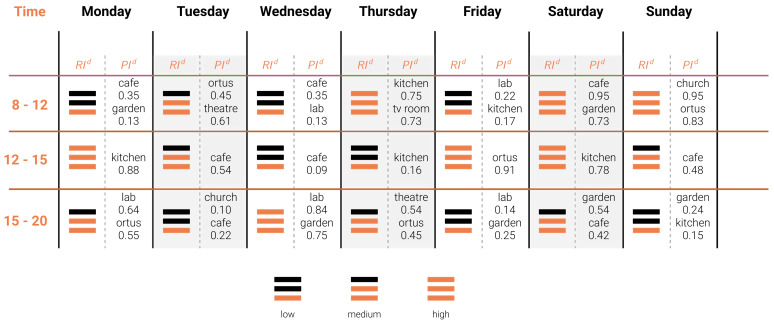
Community behaviour Prediction Table.

To support caregivers in the organization of more suitable and functional activity schedules, we built the afore-presented prediction tools for the Community RI and the PI of each place for an upcoming week. These tools require only basic data for a certain day such as the temperature, the season, the predisposition to walk on that specific day and the attribute of that day being a weekday. As such, we provide caregivers with the prediction of the Community Time-Specific Adjusted RI and the PI for each place of the village for each day of an upcoming week, thus allowing them to combine it with their knowledge to build an even more functional schedule in advance. We propose the Community behaviour Prediction Table (CBPT) (see Figure 5), a visual tool combining the predictive models to support caregivers in organizing activities at *Il Paese Ritrovato*.

This table provides the predicted value of the Community Time-Specific RI and the PI for each place for three times of the day: morning (8–12), noon (12–15) and afternoon (15–20). One of the possible ways to use the CBPT is to first observe the Community RI prediction and filter out the activities not suitable for its value. Then to choose the place in which to hold the activity by looking at the list of popularity for the places.

COVID-19 is an infectious disease caused by the most recently discovered coronavirus. This new virus and disease were unknown before the outbreak began in Wuhan, China, in December 2019. COVID-19 is now a pandemic affecting many countries globally [38]. The first reported case of COVID-19 in Italy dates to 30 January 2020, a couple of weeks later, on 17 February , the first cluster was identified in Lombardy, and on 8 March Lombardy was locked down: people could only go out of their houses to buy food or for proven working needs and commercial closed down. Prevention measures include wearing a mask to cover one’s mouth and nose, washing and sanitizing hands regularly, and, most importantly, keeping a considerable social distance of at least 1 m. The disease symptoms comprise those of a regular fever; besides, COVID-19 causes shortness of breath, leading to respiratory failure in the worst cases. Hospitals and care homes (should) have respected rigorously the preventive measures to avoid the spread of the disease among those who would suffer the most and are more likely to die from it. *Il Paese Ritrovato* had to put into place these measures, including limiting the accessible places to the apartments, thus enforcing social distancing among dwellers and caregivers.

We hereby briefly present the impact of COVID-19 on the community of *Il Paese Ritrovato* in terms of sociability and frequented places just before the lockdown, during and after it.

**Figure 6:**
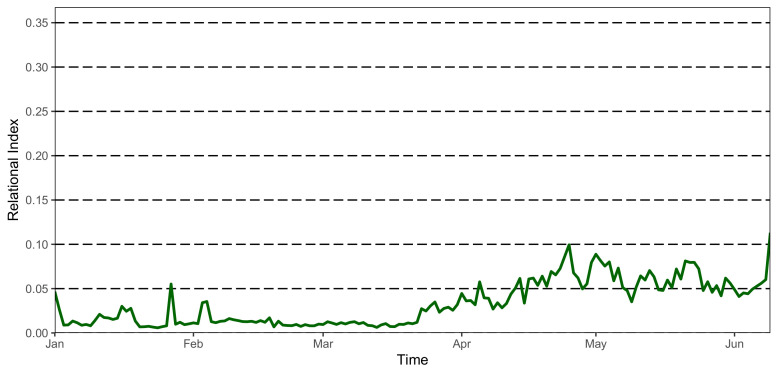
Community relational index (RI) trend during COVID-19 lockdown (January–June 2020).

We observe the Community Relational Index during the period from January 2020 to June 2020 (Figure 6).

The first period until the beginning of February is characterized by a steady low medium Community RI, probably due to the weather not being pleasant, with some peaks in correspondence of weekends, during which relatives usually visit their loved ones, so that even less sociable dwellers are encouraged by their relative to spend more time in public areas rather than alone in their room. Starting from early February the sociability at *Il Paese Ritrovato* is very low, almost 0, showing that patients were constrained to their rooms and apartments. It is only at the end of March that the sociability index starts to rise, probably thanks to the preventive measures that were solidly in place throughout the village, to the awareness of the absence of infected dwellers and staff and to the new organization allowing dwellers to spend time with one another.

Along with the observation of the Community RI, we also observe, for the same period, the PI of places in *Il Paese Ritrovato* — notice that names of places have been omitted on purpose. La Meridiana let us know that all public areas have been closed during the lockdown phase as a measure for preventing the disease from spreading, letting the dwellers stay all day inside their apartment. Hence, we decided to observe the pattern of the PI for apartments and public areas separately.

**Figure 7:**
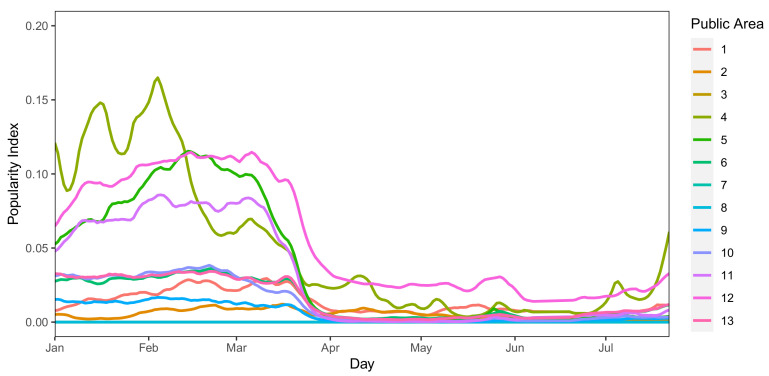
PI trend of public places at *Il Paese Ritrovato* (January–June 2020).

For all public areas of the structure, starting from the middle of March, we observe a sharp drop in the PI of these places (see Figure 7). These results are clearly in line with the COVID-preventing measures adopted and declared by *Il Paese Ritrovato*. We can also observe a timid increase in a few public areas at the end of the observation period, thus showing a relaxation of the measures also inside the structure.

**Figure 8:**
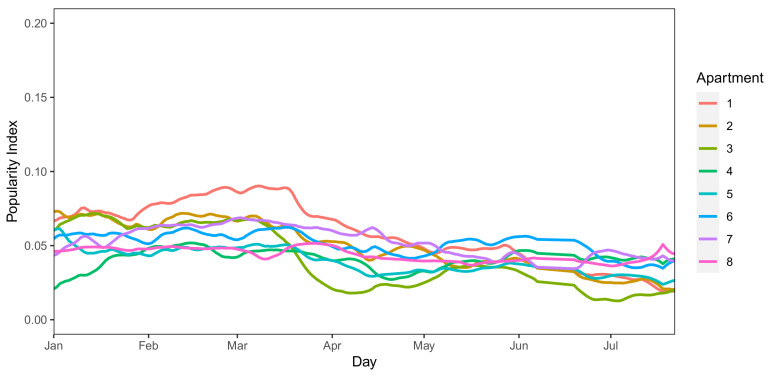
PI trend of private places at *Il Paese Ritrovato* (January–June 2020).

As far as apartments are concerned, we notice that the PI of these places is essentially constant during the whole observation period (see Figure 8 — also here, names of places have been omitted on purpose). This fact can also justify the unexpected increase in the Community RI from mid-March to June (see Figure 6) — since dwellers are forced into their apartments, sharing common areas such as corridors, TV rooms and kitchens may encourage them to socialize with their flatmates as they are the only ones they can interact with, thus increasing both the PI of these places and their RI, with a consequent increase in the Community RI as well.

In conclusion, as the preventive measures are put in place in mid-March, we observe a significant decrease in the Popularity Index of public areas, which captures gatherings and is about three times higher during normal times. On the other hand, the PI of apartment common areas is steady, as dwellers were allowed to share these areas with their flatmates, resulting in an increase in the Community RI from the end of March, after the first period of strict lockdown.

The connections in terms of social interactions between the data at our disposal allow for the use of graph representations. This form allows us to examine many aspects of the village related to the COVID-19 pandemic. La Gatta et al. [39] describe a way to predict the spread of the epidemic at regional and provincial level with neural networks. However, adapting the methods used to our case, where a micro village is considered, may not be trivial and result in poor performance.

## 4. Discussion

In this work, we presented extensive analyses, predictive tools and applications, building upon previous work [27] to further model and visualize the social behaviour of the residents of *Il Paese Ritrovato*, and to predict it to enhance social activities scheduling, with the ultimate aim of providing the residents with a better quality of life. The localization data we use is collected by a system [13] based on iBeacon technology comprising two components: a network of antennas scattered throughout the facility and a Bluetooth bracelet worn by the patients.

We extended the notion of RI to time-specific measures, rewarding a few long interactions more than many occasional encounters. We outlined the social profile of residents, identifying the way they spend their day at *Il Paese Ritrovato*. Moreover, we identified close friendships among dwellers and represented the sociability of the community through a graph showing the social network of the village.

Furthermore, we provided details on the methodology used to identify popularity trends of the places of the village. This analysis, combined with FPCA, allowed us to identify the overlooked places—seldom frequented and in need of enhancements. We also outlined sensory maps, capturing the subjective aspects of the village stimulating senses and memory.

We presented predictive tools for the estimation of both the RI and the PI, and measured their performance, which is good for the use case. These tools are of great help to the caregivers and staff of the village who can adapt activity schedules and treatments to the predicted sociability, besides being able to identify socially isolated patients. The predictions are combined into the Community behaviour Prediction Table (CBPT), a tool to visualize the estimated values of sociability and popularity of places inside *Il Paese Ritrovato*, and allowing caregivers to plan social activities accordingly.

The CBPT can be leveraged by analyzing the social activities carried out at *Il Paese Ritrovato*, such as doll and pet therapy, green care and activities involving music, both in terms of popularity and relational indexes. For instance, music is a great way to stimulate the senses and memories of patients as it stimulates reminiscence, it is not expensive, it is safe and it can be easily implemented. Music therapies have been shown to have many beneficial outcomes, including providing frameworks for meaningful activity and stimulation, management of problematic behaviour such as agitation, improved activity participation, social interaction, and social, emotional and cognitive skills. It is also shown that it lessens the feeling of being isolated [40]. Although larger places offer more space to big groups, smaller places happen to be more engaging for music-related activities. Any public space with no particular physical constraint is suitable for activities involving music. Moreover, as it stimulates participation in other activities, music could be a great way to stimulate the community to be more sociable when the relational index is low. Anyway, music can always be used as a support, played in the background while the dwellers perform other activities.

To evaluate the social distancing measures put into place during the lockdown due to COVID-19, we conducted our analysis specifically during the period from January to July 2020. We observed that social distancing was fully respected, especially during the critical months of February and March, and that starting from the end of March sociability starts to go back to normal, with a substantial increase at the end of April. This was probably due to the internal lockdown of patients in their apartment but also thanks to warmer temperatures and to the more enjoyable season. Finally, the measures implemented during the lockdown phase were perfectly captured through the PI of places, where we observe a significant drop for all public areas inside the structure.

The CBPT can be extended to a more powerful tool to promote active aging through the daily proposal of new engaging activities tailored to the preferences of residents in a user-centred perspective. Unfortunately, our lack of medical, psychological and domain-specific knowledge does not allow us to design a proper and complete dynamic activity scheduler. Nonetheless, we plan to collaborate with La Meridiana to design a more complete and powerful tool.

The introduced indexes, analyses and tools implemented at *Il Paese Ritrovato* could have great potential if applied in other facilities, possibly serving many other purposes. The possibilities offered by the same bundle—Bluetooth bracelet and network of antennas—can be suitable for several other cases. Considering the health crisis the world is experiencing due to COVID-19 and the impact it has had especially on nursing homes, the same system could be used, for instance, to monitor gatherings and trace infections.

Besides data on the movements of patients inside the facility, several other information could be included on this platform, including patient-specific medical records, treatments, and activity attendance. Most importantly, this application would allow the visualization of all the measured indexes through graphs and the usage of the Community behaviour Prediction Table.

Furthermore, a mobile application can be developed for caregivers to manage and keep an eye on their patients in a smarter way. Besides being a mirror of the afore-presented portal, the main functionalities of this application would also include alerts for anomalies in the behaviour of patients (e.g., when patients are in forbidden places such as next to the exit), an internal staff chat to avoid using third-party services for privacy reasons, an activity attendance monitoring system, and a survey-based check-up system for patients health and disease progression monitoring. This application would empower caregivers relieving them of part of the burden they experience daily.

To effectively monitor patients’ wellbeing and their health condition, other data may be collected such as the heart rate, the temperature and the sleeping activity. Smart bracelets, such as those of Empatica [41], can be used to extend data collection integrating the existing localization monitoring system. Being able to observe not only their position but also other variables could allow a full digitalization of the patient profile for enhanced data analysis.

## Figures and Tables

**Table 1:** Example of $V_{i,j}^{d}\left(t\right)$ and $M_{i,j}^{d,k}\left(t\right)$.

$V_{i,j}^{d}\left(t\right)$	1	1	0	0	1	1	1	0	0	0	0	1	1	1	1	1	1	1	1	1	…
$M_{i,j}^{d,11}\left(t\right)$	*1*	*1*	*0*	*0*	*1*	0	0	0	1	1	1	1	1	1	1	1	1	1	1	1	…
$M_{i,j}^{d,5}\left(t\right)$	*1*	*1*	1	1	1	1	1	0	0	0	0	1	1	1	1	1	1	1	1	1	…

## Data Availability

Not applicable.

## Abbreviations

The following abbreviations are used in this manuscript:

AAL	Ambient Assisted Living
AD	Alzheimer’s Disease
ALZGAR	Alzheimer’s Garden
BLE	Bluetooth Low Energy
CBPT	Community behaviour Prediction Table
PI	Popularity Index
RI	Relational Index
